# Development of a point-of-care-testing system for procalcitonin

**DOI:** 10.1186/cc11776

**Published:** 2012-11-14

**Authors:** H Kim, ES Yoon, S Ho, SW Oh, EY Choi

**Affiliations:** 1 Boditech Med Inc., Chuncheon, Korea; 2 Hallym University, Chuncheon, Korea; 3 Chonbuk National University, Jeonju, Korea

## Background

Sepsis is a daily challenge in ICUs. Today various therapeutic strategies are known to improve survival in patients with sepsis. Early assessment is important for determination of the appropriate treatment. PCT has been proposed as a specific infection maker for this purpose.

## Methods

A fluorescence dye-incorporated immunochromatographic assay (FICA) was developed for detection of the PCT concentration in serum. Serum mixed with fluorescence-conjugated detector was loaded onto a cartridge and incubated for 12 minutes. The fluorescence intensity of the cartridge was then measured in a laser fluorescence scanner. The analytical performance of the FICA system was evaluated by precision and recovery tests. The comparability of the new method was examined with an automated analyzer.

## Results

A reliable correlation between area ratio (AT/AC), reflecting fluorescence intensity of test/control line, and PCT concentration was observed (*r *= 0.999). The CVs of intra-assay and inter-assay precision in a range of 0.26 to 2.09 ng/ml were 7.8% and 4.5%, respectively, and analytical recovery of the FICA system fell within 6.4% at the tested samples. When the FICA method was compared with Roche Elecsys analyzers, there were strong correlations (*r *= 0.993, *n *= 80).

## Conclusion

The FICA system with point-of-care-testing appeared to be an easy, fast and suitable method for measurement of the PCT concentration in serum.

